# Reasons for Permanent Tooth Extractions in Japan

**DOI:** 10.2188/jea.16.214

**Published:** 2006-09-04

**Authors:** Jun Aida, Yuichi Ando, Rahena Akhter, Hitoshi Aoyama, Mineo Masui, Manabu Morita

**Affiliations:** 1 Department of Preventive Dentistry, Division of Oral Health Science, Hokkaido University Graduate School of Dental Medicine.; 2 National Institute of Public Health.; 3 Tochigi Prefectural Medical and Social Welfare College.; 4 Kanagawa Dental Association.

**Keywords:** Tooth Extraction, Tooth Loss, Epidemiology, Dental Caries, Periodontal Disease

## Abstract

**BACKGROUND:**

There has been no nationwide study in Japan on reasons for extraction of permanent teeth. This survey was aimed to determine the reasons for extraction of permanent teeth in Japan.

**METHODS:**

Five thousand, one hudred and thirty-one dentists were selected by systematic selection from the 2004 membership directory of the Japan Dental Association. The dentists selected were asked to record the reason for each extraction of permanent teeth during a period of one week from February 1 through 7, 2005. Reasons for tooth extraction were assigned to five groups: caries, fracture of teeth weakened by caries or endodontics, periodontal diseases, orthodontics, and other reasons.

**RESULTS:**

A total of 2,001 dentists (response rate of 39.1%) returned the questionnaires, and information on 9,115 extracted teeth from 7,499 patients was obtained. The results showed that caries and its sequela (totally 43.3%, 32.7% and 10.6%, respectively) and periodontal disease (41.8%) were the main reasons for teeth extraction. Extraction due to caries or fracture was commonly observed in all age groups over 15 years of age, whereas periodontal disease was predominant in the groups over 45 years of age.

**CONCLUSIONS:**

Most of the permanent teeth were extracted due to caries and its sequela and periodontal disease. Prevention and care for dental caries for all age groups and periodontal disease for over middle age groups are required.

Most of oral diseases are not critical but they are widespread. National cost of dental care took over 8% in national cost of all medical expenditure in 2002.^1^ The final consequence of most of oral diseases is tooth loss. Decrease in the number of teeth results in poor dietary habit and deterioration of quality of life (QOL).^2^ Therefore, it is important to know the reason for permanent tooth extraction. Then, information on the reasons for permanent tooth extraction will help us to plan adequate dental health policies.

Nationwide surveys to determine the reasons for extraction have been carried out in several countries.^3^^-^^12^ The main reasons for permanent tooth extraction differ among countries. Studies in many countries and some studies in small areas have shown that dental caries is the most common reason for tooth extraction.^3^^-^^8^^,^^13^^-^^19^ Proportions of Caries and periodontal disease are almost the same in Italy and Singapore,^9^^,^^10^ and periodontal disease is the most frequent reason for tooth extraction in Germany and Canada.^11^^,^^12^ Dental caries and its sequela is generally the main cause of tooth loss in young people, whereas periodontal disease is the main cause of tooth loss in middle-aged and elderly people.^3^^,^^4^^,^^7^^-^^19^ Orthodontic reason is principal reason in people younger than 20 years old.^3^^,^^4^^,^^7^^-^^19^

Morita et al^13^ reported that caries was the most frequent reason (55.4%) and periodontal disease the second-most frequent reason (38.0%) for tooth extraction in Japan. However, these data were obtained from only one prefecture of Japan and almost two decades have passed since that report. Therefore, there might have been a change in the reasons for tooth extraction.

There has been no nationwide study in Japan on reasons for extraction of permanent teeth. This nationwide survey was designed to determine the reasons for extraction of permanent teeth in Japan.

## METHODS

The study protocol was approved by the Ethical Committee of the National Institute of Public Health (Ethics Approval No. NIPH–IBRA #05003, February 28, 2005). A list of general dental practitioners in Japan (57,589 dentists) was obtained from the 2004 membership directory of the Japan Dental Association. Every tenth dentist on the list was systematically selected for the study population. In the members list, the sampling interval does not coincide with the cycle of variables. Orthodontic or pediatric dental practitioners as well as general practitioners were included. Dentists working at general hospitals on the list were also included. Then 627 dentists who had participated in the other national survey^20^ were excluded. Finally, a total of 5,131 dentists were selected from Japan’s general dental practitioners. Each dentist was sent a data collection form with a covering letter requesting that the dentist complete the form when extraction of one or more permanent teeth was carried out during one working week from February 1 through 7, 2005. Items to be filled out in the form consisted of age and sex of the dentist, the number of dental staff members in the clinic, the number of patients and working hours per day, and information on permanent tooth extraction.

Items in the questionnaire on tooth extraction consisted of age and sex of the patient and main reason for the extraction. The main reason for tooth extraction was selected from the following: (1) caries, (2) fracture of teeth weakened by caries or endodontics, (3) periodontal disease, (4) orthodontics, and (5) other reasons including third molar extractions, prosthetic reasons, and so on. The document for instructing respondents to select one reason was also attached.

Completed questionnaires were coded and data analyses were performed using the STATA^®^ 8.0 program (Stata Corporation, College Station, Texas, USA). Frequency distributions of age and sex of the respondents and those of population (all the members of the Japan Dental Association) were compared. Frequencies of reasons given by the dentists were compared according to age, sex, and tooth type. The reasons for tooth extraction were also analyzed for each tooth type in the upper and lower arches. Chi-square tests were used to compare the frequency distributions.

## RESULTS

A total of 2,001 dentists responded (recovery rate of 39.1%). Table 1 shows the distributions of the population (all the members of the Japan Dental Association) and the respondents according to age and sex. There was no statistically significant difference between the sex distributions of respondents and the population (p>0.05). The age distribution was significantly different between the two groups (p<0.001). The mean ages of the respondents and the population were 50.7 (standard deviation [SD] = 9.4) years and 51.1 (SD = 11.9) years, respectively. The mean numbers of dentists and dental hygienists were 1.3 (SD = 0.8) and 1.5 (SD = 1.4). The respondents treated about 24 patients every day, and their mean working hours were 8 hours per day.

**Table 1.** Comparison of the population and respondents according to sex, age, and number of dental staff.

		Population*	Respondents
		(n=57,784)	(n=2,001)
Sex	Male	92.8 (%)	94.0 (%)
	Female	7.2	6.0

Age (yr)	<35	5.1 (%)	2.5 (%)
	35-39	11.5	6.8
	40-44	17.7	14.8
	45-49	18.7	17.1
	50-54	16.5	16.5
	55-59	10.0	13.6
	60-64	6.2	7.1
	65-69	5.0	5.1
	70-74	4.4	1.3
	>74	4.8	1.3
	unknown	–	13.7

Mean number of dental staff			
Dentists		1.3	1.3
Dental hygienists		0.9	1.5

* Total members of the Japan Dental Association

The age of the patients ranged between 5 and 96 years. Table 2 shows the numbers of extracted teeth according to age group and sex. Totally, 9,115 teeth were extracted from 7,499 patients in the week. The differences between male and female were shown (p<0.001). Of the teeth removed, 4,610 were removed from males (mean of 1.27 extractions per male patient) and 4,505 teeth were extracted from females (mean of 1.22 extractions per female patient). The largest number of teeth extracted was in the 55-64 years of age. Females younger than 45 years in age had more extractions than did males younger than 45 years in age. In the 45-74 years of age, there were more extractions in males.

**Table 2.** Numbers of extracted teeth according to age and sex.

Age (yr)*	Male	Female	Total	%
5-14	22	30	52	0.6
15-24	204	302	506	5.6
25-34	468	476	944	10.4
35-44	371	399	770	8.4
45-54	755	703	1458	16.0
55-64	1257	1085	2342	25.7
65-74	1040	1017	2057	22.6
75-	493	493	986	10.8


Total	4610	4505	9115	100

* Age of subjects who received tooth extraction

Table 3 shows the distribution of reasons for tooth extraction. A total of 3,955 teeth (43.4%) were extracted due to caries (2,985 teeth, 32.7%) or fracture caused by caries or endodontics (970 teeth, 10.6%), 3,812 (41.8%) teeth were extracted due to periodontal disease, and 1.2% of teeth were extracted due to orthodontics. Others (13.6%) were extracted because of third molar removal (797 teeth) or prosthetic reasons. The differences between male and female were also shown (p<0.001). The percentages of caries extraction were 32.0% in males, and 33.6% in females, and more teeth were extracted because of periodontal disease in males (46.1%) than in females (37.4%).

**Table 3.** Numbers and percentages of tooth extractions according to reason.

Reason	Male		Female		Total	
		
	n	%	n	%	n	%
Caries	1473	32.0	1512	33.6	2985	32.7
Fracture	438	9.5	532	11.8	970	10.6
Periodontal disease	2126	46.1	1686	37.4	3812	41.8
Orthodontics	25	0.5	86	1.9	111	1.2
Others	548	11.9	689	15.3	1237	13.6
Total	4610	100	4505	100	9115	100

**Figure 1.**
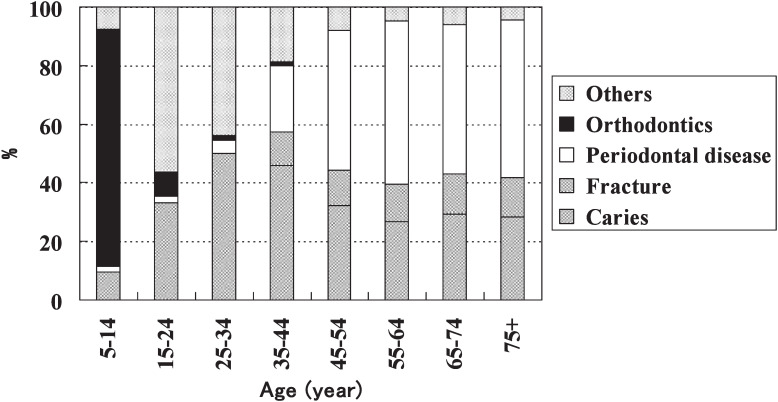
Distribution of the reasons for tooth extraction according to age.

Figure 1 shows the reasons for tooth extraction according to age. Extraction due to caries or fracture was commonly observed in all age groups over 15 years. Caries or fracture was the reason for extraction in 50.1% and 57.3% of the patients in the 25-34 years and 35-44 years age groups, respectively. However, the percentage of patients who underwent extraction for periodontal disease was predominant in the 45-54 years and older. The main reason for extraction in patients under 15 years of age was orthodontics (in 80.8% of patients 5-14 years of age). In the 15-24 year age group, other reason was predominant, indicating the high percentage of third molar tooth extraction.

**Figure 2.**
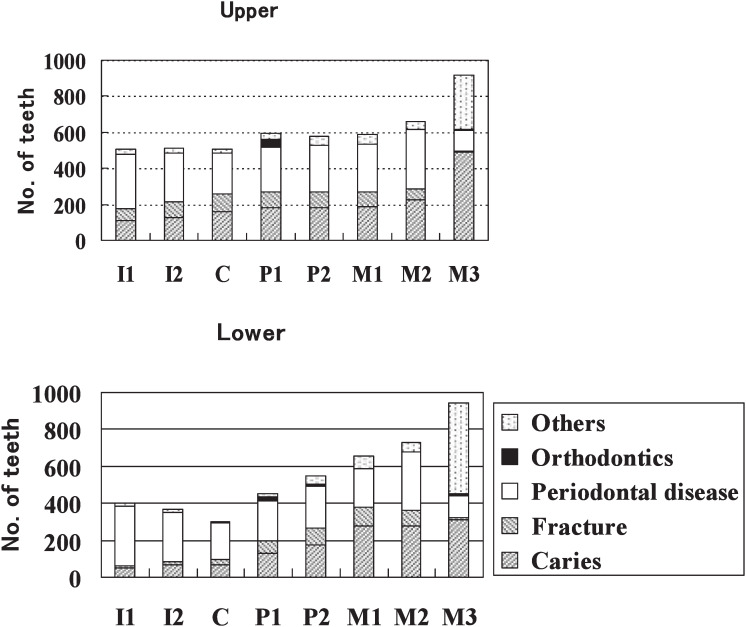
Distribution of the reasons for tooth extraction according to tooth type. I1: Central incisor, I2: Lateral incisor, C: Canine, P1: First premolar, P2: Second premolar, M1: First molar, M2: Second molar, M3: Third molar

Data were not divided into right and left quadrants, because there was no difference between rates of extraction on the left and right sides of the oral cavity (p>0.05). Regarding individual teeth, maxillary and mandibular third molar removal was observed most frequently (919 and 946 teeth, respectively). Seventy-six point six percent of the central and lateral incisors in the mandible were extracted for periodontal disease (Figure 2). The percentages of extraction due to fracture were the highest for the canine tooth in the maxilla (18.8%) and for the 2nd premolar in the mandible (17.0%).

## DISCUSSION

There have been some studies on the reasons for tooth extraction in Japan,^13^^,^^21^ but all of them targeted subjects in local areas. For example, a study in 1986-1987 targeted 849 dentists in one prefecture of Japan, and the response rate was 38%.^13^ The present study is the first nationwide study on reasons for tooth extraction in Japan.

There are several limitations when interpreting the data. At first, the information of this survey was obtained using outpatients in dental clinic because we assumed majority of tooth loss occurred in dental clinic. Therefore, it was difficult to estimate precise prevalence of tooth loss. Secondly, the samples were selected from the 2004 membership directory of the Japan Dental Association, and.the non-members of the Japan Dental Association did not participate in the present study. In addition, there was a statistically significant difference between the age distributions of respondents and the total members of the Japan Dental Association. Therefore, the selected samples and the respondents are not necessarily representative of the dentists in Japan. The mean number of dental hygienists employed by the respondents was higher than that in the representative dental clinics (Table 1). It is possible that only dentists working with the sufficient numbers of co-dental staff responded to our questionnaire. Tertiary, although the response rate in our study (39.1%) was similar to response rate in previous surveys,^7^^,^^9^^,^^12^^,^^17^ this value was not high. The reinforcement of participation, i.e., asking by telephone or mail, might have increased the response rate. However, we could not carry out these efforts because of the financial problem and limited man-power.

The results of the present study showed that 32.7% of the teeth were extracted due to caries and 10.6% were due to tooth fracture. Because “fracture” means a situation in which a tooth has been weakened by caries or endodontics, it is reasonable to assume that fracture is indirectly related to caries. In previous studies, fracture was considered as a sequela of caries.^3^^-^^9^^,^^13^^-^^18^ Trovik et al^3^ reported that 40.2% of the teeth extracted were due to caries and sequelae. Similar to this finding, 43.2% of the teeth extracted are because of caries and its sequela in Japan. Extraction due to caries or fracture was commonly observed in all age groups over 15 years. This result suggests that caries occurs through the life of dentulous. For example, coronal caries is prevalent among to young people and root caries among aged people.

The present study showed that 41.8% of the teeth extracted were due to periodontal disease. The previous regional epidemiologic studies in Japan showed that 38% (1994) and 46% (2001) of teeth extracted were due to periodontal disease.^13^^,^^21^ Therefore, this figure of 41.8% dose not necessary suggest the meaningful change in the rate of tooth extraction due to periodontal disease. Our study confirmed the trend that periodontal disease was the most frequent reason for tooth extraction in patients over 45 years of age as shown in previous studies.^3^^,^^4^^,^^6^^-^^19^

Natural consequence of aging does not cause severe periodontal disease, and a small proportion of persons exhibit severe periodontitis in which tooth loss occurs.^22^^,^^23^ Therefore, a large number of extractions might be extracted due to periodontal disease in a relatively small number of patients.^24^ For example, Phipps and Stevens^25^ reported that 51% of teeth extracted were because of periodontal disease and 35.4% were because of caries. However, when considering patients as the unit of analysis, 58% of patients had extraction for caries and 40% had extraction for periodontal disease. Based on these aspects, effective case-finding approach is required. However, there has been no efficient way of screening for periodontal disease in public health activities so that targeted population approach for after middle age people might be required.

The present study was the first national survey on tooth extraction in Japan. About 85% of permanent teeth extracted were extracted due to caries and its sequela, and periodontal disease. Because extraction due to caries or fracture was commonly observed in all age groups over 15 years, prevention of caries including root caries among aged people through over age should be required. Multiple teeth are often extracted in a patient with severe periodontal disease so that targeted population approach on prevention for after middle aged people in public health setting and case-finding approach for severe periodontal disease in dental clinic setting is important. Dentists should take measures to minimize tooth loss for these main reasons. Continuing and storing of such studies will support the prediction of tooth survival in Japan.
